# FAPi-PET/CT as surrogate marker for subclinical myocardial fibrosis is a prognostic tool for patients undergoing left-sided heart valve surgery

**DOI:** 10.1186/s13550-025-01324-5

**Published:** 2026-04-03

**Authors:** Theresa Holst, Alexander Dierks, Marianne Patt, Georgine Wienand, Lisa Müller, Christian H. Pfob, Malte Kircher, Sina Stock, Constantin Lapa, Evaldas Girdauskas, Ralph A. Bundschuh

**Affiliations:** 1 Department of Cardiothoracic Surgery, Faculty of Medicine, Augsburg University Hospital, Augsburg, Germany; 2 Department of Cardiothoracic, Transplantation and Vascular Surgery, Hannover Medical School, Hannover, Germany; 3 Department of Nuclear Medicine, Faculty of Medicine, Augsburg University Hospital, Augsburg, Germany; 4 Department of Nuclear Medicine, University Hospital Carl Gustav Carus at the TU Dresden, Fetscherstr. 74, Dresden, 01307 Germany

**Keywords:** FAPi-PET/CT, Cardiac fibrosis, Aortic valve disease, Mitral valve disease

## Abstract

**Background:**

The aim of this study was to evaluate fibroblast activation protein (FAP)-targeted positron emission tomography and computed tomography (PET/CT) imaging as surrogate marker for cardiac fibrosis and therefore as prognostic tool for patients with left-sided valvular heart disease undergoing open-heart surgery.

**Results:**

In 13 patients (6 men, 7 women; mean age ± SD, 62 ± 13 years) scheduled for aortic and/or mitral valve surgery (aortic, *n* = 3; mitral, *n* = 9; both, *n* = 1) preoperative FAPi-PET/CT ([^68^Ga]Ga-RTX1363S) was perfomred. FAP-positive volume and FAP uptake was quantified by different methods (isocontour with fixed and variable threshold) and correlated to preoperative clinical parameters, including (left ventricular ejection fraction (LVEF) and serum N-terminal prohormone of brain natriuretic peptide (NT-proBNP), as well as to changes in LVEF from preoperatively to 3–6 months follow-up (available for *n* = 11 patients). FAP-uptake in the left ventricular (LV) myocardium was highly variable in the study cohort. Isocontour-55%-based FAP-positive volume was found to correlate statistically significantly with preoperative LVEF (*p* = 0.03) and preoperative NT-proBNP (*p* = 0.02). In addition, we found a significant correlation with the change in LVEF from preoperatively to 3–6 months follow-up examination (*p* = 0.04).

**Conclusions:**

Preoperative FAPi PET/CT seems to have potential to predict LVEF changes after valvular heart surgery and may therefore be an important tool for risk stratification of asymptomatic patients with left-sided valvular heart disease to support the appropriate timing of valvular surgery.

**Supplementary Information:**

The online version contains supplementary material available at 10.1186/s13550-025-01324-5.

## Introduction

Aortic and mitral valve disease are the two most prevalent entities of acquired valvular heart disease (VHD) in the Western hemisphere with increasing prevalence with advancing age [1]. Long-standing aortic and mitral valve dysfunction lead to increased pressure or volume overload of the left ventricle (LV). Owing to compensatory mechanisms, affected patients may remain asymptomatic for a long time [2, 3]. Yet, persisting volume and pressure overload induce concentric or eccentric LV remodeling, ultimately resulting in cardiomyopathy and progressive left-sided heart failure, if left untreated [4]. However, according to recent joint clinical practice guidelines by the European Society of Cardiology and European Association of Cardiothoracic Surgery (ESC/EACTS) [5], surgery/intervention for severe aortic valve disease is indicated only if significant LV dysfunction/enlargement is detected on echocardiography or if the patient develops clinical symptoms attributable to VHD. The treatment recommendations are more liberal in case of primary mitral regurgitation, for which surgical repair is already indicated in the absence of clinical symptoms when LV dysfunction is detected [5]. In theory, the valvular cardiomyopathy should recover after successful correction of aortic or mitral valve disease. However, in the real world, only a proportion of patients achieve satisfactory LV re-remodeling [6–8]. Postoperative persistence and progression of cardiomyopathy may be associated with an advanced, subclinical myocardial fibrosis impeding postoperative repair mechanisms and hence LV reverse remodeling [6, 8–10]. Consequently, missing LV re-remodeling after surgical/catheter-based valve treatment is associated with higher mortality [11, 12]. Surgical intervention in patients with asymptomatic left-sided valvular heart disease at an earlier time point may potentially reduce or even stop fibroblastic remodeling of the LV myocardium and therefore improve patient outcome [13]. Unfortunately, no established biomarkers are available to reliably detect and quantify subclinical myocardial damage before significant increase in LV diameter and volume occurs.

In recent years, several non-invasive, imaging-based biomarkers gained in relevance for diagnosis and treatment decisions, often resulting in improved patient outcome. Late gadolinium enhancement in magnetic resonance imaging (MRI) for differentiation of scar tissue and irreversible fibrosis in the myocardium became routine in daily clinical practice [14]. However, this technique is not able to evaluate dynamic changes in early diffuse fibrotic remodeling [15], therefore T1 mapping for quantification of extracellular volume as a surrogate parameter for diffuse myocardial fibrosis is gaining on importance [16, 17]. However, T1 mapping is highly dependent on the hardware and the scanning protocols used and, therefore, it has not yet been established in the clinical practice [16].

Another emerging imaging modality is a hybrid positron emission tomography and computed tomography (PET/CT) using a radiotracer targeting the fibroblast activation protein (FAP) [18]. Such inhibitors to the FAP (FAPi) can be implemented to visualize and quantify the distribution of activated fibroblasts. By targeting activated myocardial fibroblasts, FAPi-PET/CT specifically allow for visualization of cell activity in the early dynamic phase of LV remodeling before the burnt-out state of irreversible fibrotic scarring occurs [18]. Consequently, a reduced or low FAP-expression correlates with a late phase of the remodeling process and hence with the state of irreversible damage [19]. Thus far, however, only few reports evaluating the value of FAPi-PET/CT in cardiovascular diseases have been published [15, 20–23], including a single study on patients with aortic valve stenosis who underwent transcatheter AV replacement [24]. Herewith, we report on the value of FAPi-targeted PET/CT in patients with left-sided VHD undergoing open-heart surgery. We evaluate the potential prognostic value of FAPi-PET/CT in such patients, thereby ultimately aiming to improve risk stratification and timing of surgery.

## Methods

### Patients

13 patients (6 men, 7 women; mean age ± SD, 62 ± 13 years) scheduled for aortic and/or mitral valve surgery (aortic, *n* = 3; mitral, *n* = 9; both, *n* = 1) underwent a preoperative FAPi-PET/CT evaluation and were included in this retrospective analysis. Perioperative risk profile was low (mean European System for Cardiac Operative Risk Evaluation (EuroSCORE) II ± SD, 1.68 ± 1.29%; mean Society of Thoracic Surgeons (STS) predicted risk of mortality ± SD, 1.76 ± 2.23%). Patients were excluded if they required concomitant right-sided heart valve surgery, coronary artery bypass grafting or treatment for active endocarditis or if they had undergone previous cardiac surgery. The retrospective study was approved by the local Ethics Committee (review board of the Ludwig-Maximilians-Universität München, Munich, Germany; permit number 24–0731). All patients gave written and informed consent for the diagnostics and therapeutic procedures. The study was conducted in accordance with the Helsinki Declaration and with national regulations.

## Radiopharmaceutical preparation and imaging

[^68^Ga]Ga-RTX1363S, which was established in our department for oncological questions before [25], was prepared in-house in compliance with the German Medicinal Products Act, AMG§ 13 2b, and after informing the responsible regulatory body. The precursor RTX1363S (90 µg/dose) was provided by RatioTherapeutics (Boston, USA). The synthesis of [^68^Ga]Ga-RTX1363S was performed as automated sequence using a GRP-3 V module (Scintomics GmbH, Graefelfing, Germany) with a GMP-compliant cassette and reagents by ABX (ABX GmbH, Radeberg, Germany) with a IRE elite generator (ROTOP, Dresden, Germany). Patients were intravenously injected with in mean 163 MBq (ranging from 132 MBq to 188 MBq) [^68^Ga]Ga-RTX1363S. 42 to 64 min (in mean 51 min) after the injection patients were scanned on a GE Discovery MI PET/CT system (General Electrics, Milwaukee, USA) [26] from the vertex to the thigh (2 min per bedposition), in addition a 10 min scan over the heart was performed. PET images were reconstructed by an iterative algorithm implemented by the manufacturer (3 iterations, 8 subsets, 5 mm Gausian filter). In all patients, a low-dose CT for anatomical correlation, scatter and attenuation correction was acquired (120 mV, 16 mAs, dose modulation). Depending on the clinical question, additional diagnostic CT protocols were performed in some patients.

## PET image analysis

For analysis of the [^68^Ga]Ga-RTX1363S PET data, images acquired for 10 min over the heart were used. Several parameters were analyzed in the images: Firstly, an isocontour using a threshold of 55% of the maximum uptake in the myocardium of the left ventricle was used to define the FAP-positive volume (Isocontour-55%). Within this FAP-positive volume the maximum and mean standardized uptake value (SUV) was calculated. Secondly, an isocontour using a fixed threshold of SUV 3.5 (Isocontour-3.5) was used to calculate the FAP-positive volume, maximum and mean SUV were calculated in this volume accordingly. These two methods have been chosen as we found them to be most reliable. A background(bloodpool)-based method as performed by Diekmann and colleagues [24] was applied as well but showed very high fluctuations and could not provide a segmented volume in four of our 13 patients. In addition, we evaluated the maximum SUV by placing a volume of interest around the right ventricle without covering the septum, which is considered to be part of the left ventricle and was included in this analysis. Two board certified specialists in nuclear medicine with three- and five-years of experience in cardiovascular PET imaging, independently and blindly analyzed each [^68^Ga]FAPi-PET/CT in random order and derived parameters are given as the mean of the two observers.

## Surgical procedure

Isolated mitral valve surgery was performed through a right anterolateral mini-thoracotomy in the fourth intercostal space using a fully endoscopic 3D technique [27]. Isolated aortic valve surgery was achieved through a partial upper J mini-sternotomy in the third or fourth intercostal space [28]. Combined mitral and aortic valve surgery was performed through a median sternotomy. Valve repair was pursued in pure valvular regurgitation while valvular stenosis and mixed valve disease was treated by prosthetic valve replacement [29].

## Clinical follow-up

One patient who underwent double valve replacement surgery for combined severe aortic and mitral valve stenosis leading to relevant pressure overload and severe LV dysfunction (LV ejection fraction (LVEF) 35%) died from refractory cardiac dysfunction at two weeks postoperatively after unsuccessful weaning from extracorporal life support (i.e., arterio-venous extracorporal membrane oxygenation) required for post-cardiotomy low cardiac output syndrome (LCOS). Another patient who underwent surgery for severe mitral regurgitation causing relevant volume overload of the LV, but with preserved LVEF (55%), died from progressive LV heart failure at one month postoperatively. All surviving patients were routinely invited to a follow-up visit at our institution at 3–6 months postoperatively. Transthoracic echocardiography was performed and LVEF evaluated by an experienced senior cardiologist with substantial expertise in echocardiography. If patients presented solely to their referral cardiologist, digital transthoracic echocardiographic images were requested for further systematic analysis. Mean follow-up of all surviving patients was 4 ± 2 months.

### Statistics

Due to small sample size, reported data are mainly descriptive. All continuous data are expressed as mean, standard deviation and range. Correlations between preoperative and follow-up clinical characteristics and preoperative FAPi-PET/CT-derived data were calculated using the Pearson correlation method. The correlation coefficient *r*, the 95%-confidence interval and the significance level *p* are reported. All *p*-values < 0.05 were considered statistically significant. For graphical presentation, scatter diagrams are used. Patients for whom no follow-up at 3–6 months postoperatively was available (i.e., the two patients who deceased during early postoperative follow-up) were excluded from the correlation analyses. All statistical analyses were done using MedCalc (version 22.0.13; MedCalc Software, Ostend, Belgium).

## Results

As displayed in Table 1, left-sided VHD caused significant LV volume overload in 9 patients (69%) while 4 patients (31%) suffered predominantly from LV pressure overload. Preoperative LVEF ranged from 35 to 70% with a mean value of 54% in all patients. Preoperative NT-proBNP (i.e., N-terminal prohormone of brain natriuretic peptide) ranged from 113 to 11,400 ng/l with a mean value of 2487 ng/l. Most patients reported dyspnoea on exertion and limitation during ordinary activity to at least some degree (i.e., New York Heart Association (NYHA) class II in *n* = 7, 54%; NYHA III in *n* = 4, 31%). The preoperative data of 11 patients for whom postoperative follow-up at 3–6 months was available are presented in Table 2 and for each patient in Supplementary Table 1.

**Table 1:** Preoperative and follow-up clinical characteristics of all *n* = 13 patients

	Mean	SD	Range	n	%
**Type of VHD** - Mitral valve disease - Aortic valve disease - MV and AV disease				9 3 1	69 23 8
**Type of LV overload** - Volume overload - Pressure overload				9 4	69 31
**Preoperative NT-proBNP (ng/l)**	2487	3619	111–11,400		
**Preoperative NYHA class** - I - II - III				2 7 4	15 54 31
**Preoperative LVEF (%)**	54	11	35–70		
Postoperative LVEF* (%)	50	13	15–65		
**ΔLVEF (preop to last follow-up)**	−4	10	−20–15		

AV: aortic valve; LV: left ventricle; LVEF: left ventricular ejection fraction; MV: mitral valve; NT-proBNP: N-terminal prohormone of brain natriuretic peptide; NYHA: New York Heart Association; VHD: valvular heart disease; Δ: delta; *at last documented follow-up.

**Table 2:** Preoperative and follow-up clinical characteristics of the *n* = 11 patients included in the correlation analyses

	Mean	SD	Range	n	%
**Type of VHD** - Mitral valve disease - Aortic valve disease - MV and AV disease				8 3 0	73 27 0
**Type of LV overload** - Volume overload - Pressure overload				8 3	73 27
**Preoperative NT-proBNP (ng/l)**	1769	2666	113–8960		
**Preoperative NYHA class** - I - II - III				2 7 2	18 64 18
**Preoperative LVEF (%)**	55	10	40–70		
Postoperative LVEF* (%)	53	7	40–65		
**ΔLVEF (preop to last follow-up)**	−2	10	−20–15		

AV: aortic valve; LV: left ventricle; LVEF: left ventricular ejection fraction; MV: mitral valve; NT-proBNP: N-terminal prohormone of brain natriuretic peptide; NYHA: New York Heart Association; VHD: valvular heart disease; Δ: delta; *at last documented follow-up.

**Fig. 1:**
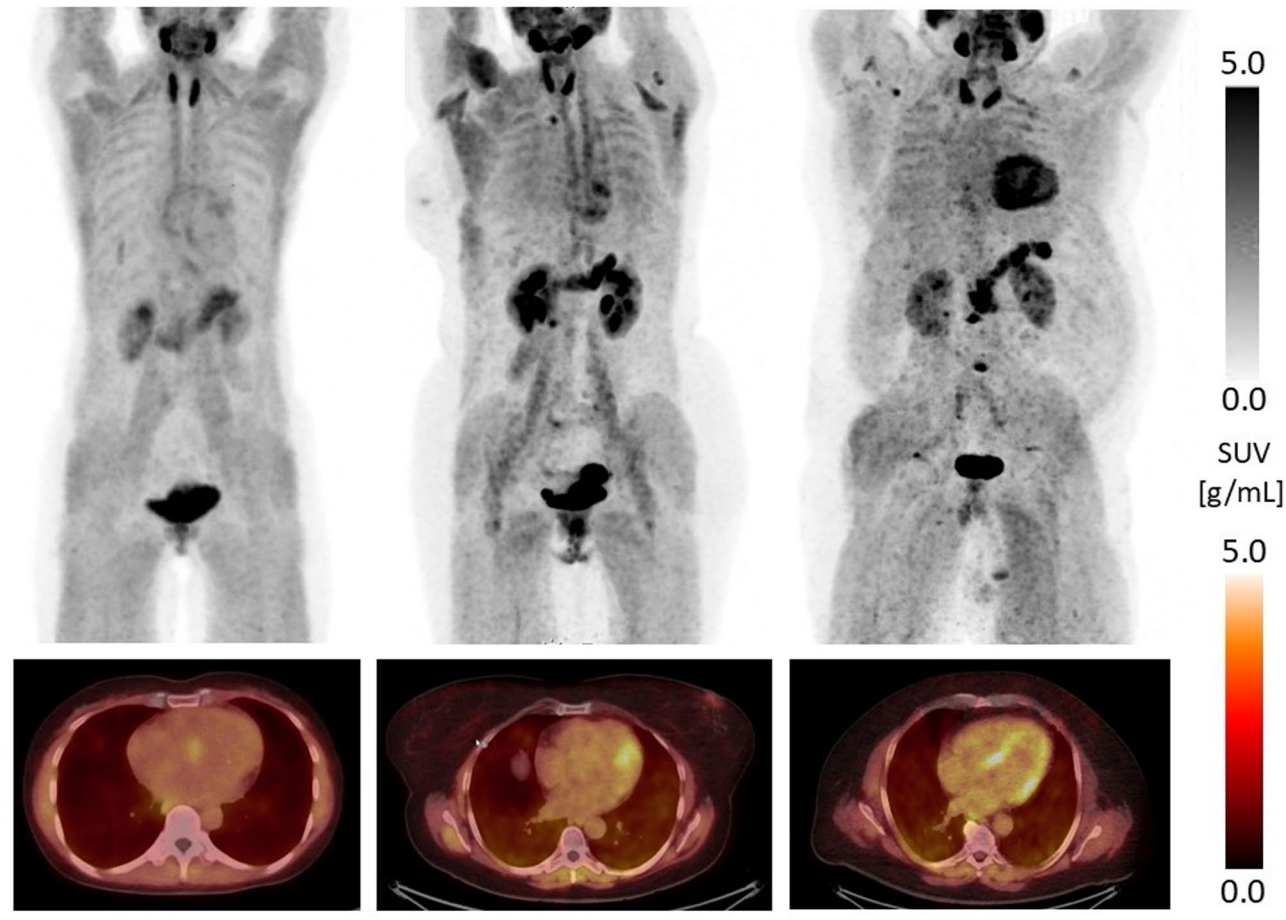
Highly variable patterns of FAP-uptake in PET maximal intension projection (upper row) and fused PET/CT images (lower row) in the left ventricular myocardium in different patients, ranging from no uptake at all (left), partial uptake in some regions (middle) to increased uptake over the whole left ventricular myocardium (right). FAP: fibroblast activation protein; SUV: standardized uptake value

[^68^Ga]Ga-RTX1363S uptake in the LV myocardium was highly variable on preoperative FAPi-PET/CT in our study cohort. As illustrated in Table 3, for Isocontour-55% maximum standardized uptake value (SUV) ranged from 3.0 to 11.3 with a mean value of 6.5 while mean SUV ranged from 1.9 to 7.8 with a mean value of 4.2. The FAP-positive volume ranged from 23 ccm to 159 ccm with a mean value of 93 ccm. In case of Isocontour-3.5, variation was even higher, especially the FAP-positive volume ranged from 0 ccm to 491 ccm with a mean value of 114.0 ccm and a standard deviation of 142 ccm. Patient individual uptake values can be found in Supplementary Table 1. As shown in Fig. 1, uptake pattern was highly divergent in our study cohort ranging from almost no relevant uptake to marked diffuse uptake in the whole LV myocardium. Corresponding FAPi-PET/CT data for the *n* = 11 for whom follow-up at 3–6 months postoperatively was available is reported in Table 4. No relevant FAP-uptake could be found in the right ventricular myocardium in our cohort, neither hotspots could be identified visually. The maximum SUV in the right ventricle was in mean 3.1 ranging from 1.9 to 5.1 and therefore significant (*p* < 0.05) lower than in the left ventricle myocardium.

**Table 3:** Preoperative FAPi-PET/CT data for all *n* = 13 patients

	Mean	SD	Range
**Isocountour-55%**			
** Maximum SUV**	6.5	2.7	3.0–11.3
** Mean SUV**	4.2	1.8	1.9–7.8
** FAP-positive volume (ccm)**	93	39	23–159
**Isocontour-3.5**			
** Maximum SUV**	6.5	2.7	3.0–11.3
** Mean SUV**	4.2	0.5	3.7–5.0
** FAP-positive volume (ccm)**	114	142	0–491

FAP: fibroblast activation protein; SUV: standardized uptake value.

**Table 4:** Preoperative FAPi-PET/CT data for the *n* = 11 patients included in the correlation analyses

	Mean	SD	Range
**Isocountour-55%**			
** Maximum SUV**	7.1	2.5	3.9–11.3
** Mean SUV**	4.6	1.7	2.5–7.8
** FAP-positive volume (ccm)**	89	40	23–159
**Isocontour-3.5**			
** Maximum SUV**	7.1	2.5	3.9–11.3
** Mean SUV**	4.3	0.5	3.7–5.0
** FAP-positive volume (ccm)**	135	146	1–491

FAP: fibroblast activation protein; SUV: standardized uptake value.

At 3–6 months postoperatively, LVEF ranged from 40 to 65% with a mean value of 53% in all surviving patients. Compared to preoperatively, mean change in LVEF was − 2% with a range of −20 to + 15% (see Table 2).

The two patients who died early after surgery due to persistent LCOS showed a moderately increased FAP-uptake (maximum SUV of 4.0 and 3.0) with a FAP-positive volume of 130 ccm and 100 ccm, respectively (Isocontour-55%).

## Correlation analysis between FAPi uptake and severity of valvular cardiomyopathy

**Fig. 2:**
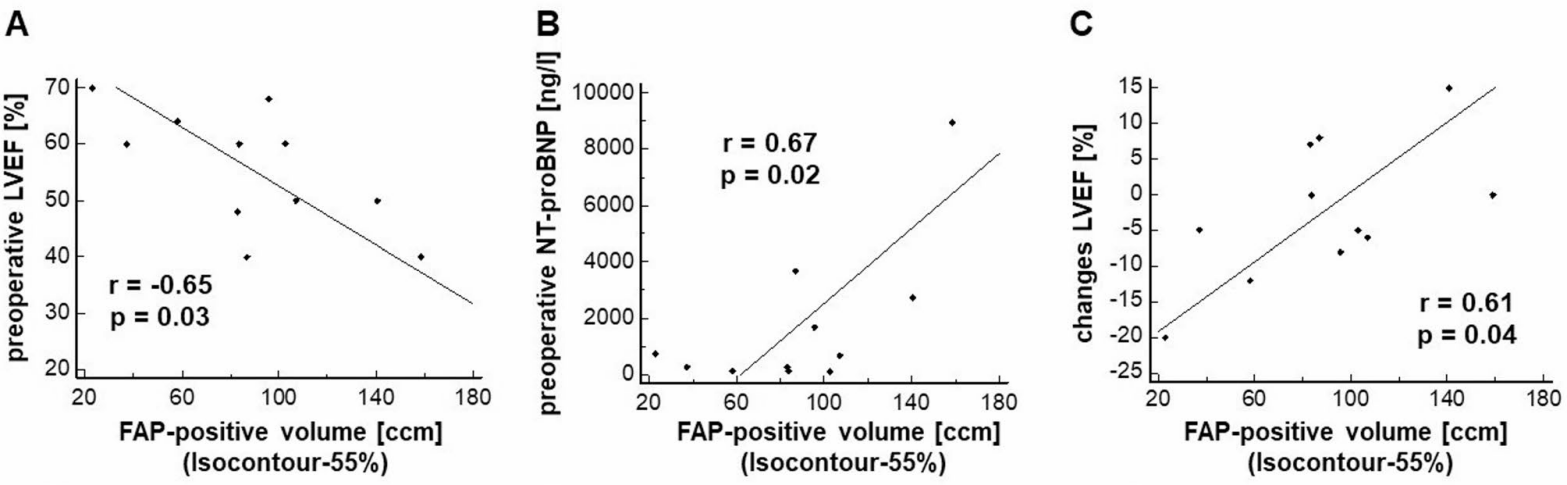
Relationship between preoperative FAP-positive volume (Isocontour-55%) and preoperative LVEF (**A**), preoperative NT-proBNP value (**B**) and changes in LVEF from preoperatively to 3–6 months postoperatively (**C**). FAP: fibroblast activation protein; LVEF: left ventricular ejection fraction; NT-proBNP: N-terminal-pro brain natriuretic peptide

Preoperative FAP-positive volume (Isocontour-55%) showed a significant negative linear correlation with preoperative LVEF (*r* = − 0.65; *p* = 0.03) (see Fig. 2A). Furthermore, FAP-positive volume correlated significantly with preoperative NT-proBNP value (*r* = 0.67; *p* = 0.02) (see Fig. 2B). All other parameters (including maximum and mean SUV) did not show any significant correlation, especially not the parameters measured using the Isocontour-3.5.

### Correlation analysis between FAPi uptake and postoperative LV re-remodeling

We found a significant positive linear correlation of the preoperative FAP-positive volume obtained by Isocontour-55% and change in LVEF from preoperatively to 3–6 months postoperatively (*r* = 0.61; *p* = 0.04) (see Fig. 2C). The activity uptake parameters, however, did not show any significant correlation (SUV_max_: *r* = − 0.01, *p* = 0.97; SUV_mean_ Isocontour-55%: *r* = − 0.06, *p* = 0.86; SUV_mean_ Isocontour-3.5: *r* = 0.11, *p* = 0.76), neither did the FAP-positive volume defined by Isocontour-3.5 (*r* = 0.29, *p* = 0.39).

## Discussion

Our study aimed to explore the value of FAPi-PET/CT-derived imaging parameters as a potential novel imaging biomarker in patients with severe left-sided VHD scheduled for open heart surgery. We observed highly heterogeneous preoperative FAP uptake in terms of standardized uptake value (SUV) and FAP-positive volume in the LV myocardium. Our findings are in accordance with the recently published study on patients with aortic valve stenosis scheduled for transcatheter aortic valve replacement [24]. We hypothesize that the heterogeneity in FAP-uptake in our study cohort reflects the different stages of myocardial fibroblast activation and, hence, a wide range of subclinical LV remodelling. We found that the preoperative FAP-positive volume, calculated by a variable threshold based on the maximum uptake in the myocardium, correlated significantly with the preoperative LV function as well as with levels of preoperative serum NT-proBNP, which is consistent with the previous results of Diekmann and colleagues [24]. In contrast to our study, echocardiographic follow-up in the abovementioned study ended at the time of hospital discharge and the authors observed a significant correlation between FAP-positive volume and short-term postinterventional improvement in LVEF [24]. We followed our study cohort up at 3–6 months postoperatively to allow for recovery from early postoperative transient LV systolic function decline that is common after resolution of LV volume and pressure overload after open heart valve surgery [30, 31]. We found a significant inverse correlation between FAP-positive volume and improvement in LVEF from baseline to mid-term follow-up. This finding is somehow counterintuitive and suggests that patients with a higher preoperative FAP-positive volume (i.e., a greater number of activated fibroblasts and, hence, most likely more ongoing subclinical remodelling) experienced the most intense improvement in LVEF at 3–6 postoperatively. In other words, the more intense FAPi uptake was associated with a higher chance of postoperative LV functional recovery. There might be several potential explanations for an inverse association between FAPi uptake volume and the systolic LVEF change at 3–6 months after heart valve surgery. First of all, low preoperative FAP-positive volume was predominantly found in patients with a preserved baseline LVEF who cannot reach any further improvement in LVEF postoperatively. Moreover, those patients with an advanced LV dysfunction may exhibit a burnt-out end-stage fibrotic LV remodelling that coincides with a low residual fibroblast activity. Such patients would also show low FAP-expression and simultaneously lack the potential for LV functional recovery postoperatively. This statement is further supported by the previously published evidence [19]. In this paper Songe and colleagues found in a preclinical study in a heart failure rat model, that after initial increasing FAP-uptake, it decreased over time as cardiac fibrosis and the degree of myocardial injury increased. The authors conclude from their results, that FAP-uptake is high in early stages of heart failure and then decreases when remodelling is completed to an end-stage fibrosis. For analysis of FAPi-PET, we found that pure uptake alone is not an optimal parameter for clinical correlation while FAP-positive volume revealed significant correlation, in line with the study by Diekmann and colleagues [24]. In addition, our findings suggest, that a volume delineation based on a variable threshold is more suitable than a fixed threshold, which is obvious when considering the high variability of FAP expression between different patients. A variable threshold based on the background activity, respectively the blood pool activity as applied by Diekmann et al. [24], did not show a reliable result in our cohort, as in four of the 13 patients no segmentation could be performed at all.

Two of the patients in our cohort died before the follow-up examination. The first of these patient with a normal EF of 55% before surgical intervention and an moderate FAP-uptake (SUVmax 3.96) but a high FAP-positve volume of 130 ccm and an extrem high NT-proBNP of 11,400 ng/ml died about 5 weeks after the surgical intervention in acute heart failure, even FAP-uptake and volume may suggest, that remodelling was still in progress and surgical and the patient may benefit from surgical intervention. The second patient died two weeks after surgical intervention and despite exploitation of all intensive care option in acute heart failure. In this patient EF was already reduced before intervention with EF of 35% and FAP-uptake was moderate to low with SUVmax 2.97, so it may be speculated that the fibrotic process was already well advanced with less active remodelling.

Our preliminary findings should be validated by subsequent, larger-scale, prospective studies, including patients with the whole spectrum of valvular cardiomyopathy. When confirmed by subsequent studies, [^68^Ga]FAPi-PET/CT may have important an clinical implication in the future decision-making process. In particular, [^68^Ga]FAPi-PET/CT could be potentially used for improved risk stratification of asymptomatic patients with left-sided VHD to support the appropriate timing of valvular surgery.

### Limitations

Our findings should be considered as hypothesis-generating and preliminary due to an observational study design and small sample size. Moreover, recruitment of patients across the entire disease spectrum was hampered by the fact that decompensated patients with severely reduced preoperative LVEF at the initial diagnosis of left-sided VHD were generally referred for urgent surgery. The time for extensive diagnostic work-up, including preoperative FAPi-PET/CT examination, was limited in such patients. In addition, we included a heterogeneous patient cohort with left-sided valvular lesions leading to volume overload as well as pressure overload. A technical limitation is for sure the method how to segment the FAP-positive volume, which was chosen empirically in our patients. However, there is currently no established method, so we have chosen a variable as well as a fixed threshold for segmentation for our analysis, which are typical methods in PET segmentation in general [32]. Another major limitation for sure is, that based on the clinical workflow and capacity, unfortunately no magnetic resonance imaging (MRI) examination were performed before surgery in these patients, so we could not do any comparison to MRI data.

## Conclusion

In conclusion, preoperative FAPi-PET/CT imaging reveals highly heterogeneous myocardial uptake patterns in patients with severe left-sided VHD. Preoperative FAPi-positive volume correlates significantly with the LV functional recovery after left-sided valvular heart surgery. Subsequent prospective studies are necessary to validate the value of FAPi-PET/CT examination in the clinical decision-making process in asymptomatic patients with left-sided VHD.

## Supplementary Information

Supplementary Material 1

Supplementary Material 2

Supplementary Material 3

## Data Availability

The data sets generated during and/or analyzed during the current study are available from the corresponding author upon reasonable request.

## References

[1] Iung B, Vahanian A. Epidemiology of valvular heart disease in the adult. Nat Rev Cardiol. 2011;8(3):162–72. 21263455 10.1038/nrcardio.2010.202

[2] Dweck MR, Boon NA, Newby DE. Calcific aortic stenosis: a disease of the valve and the myocardium. J Am Coll Cardiol. 2012;60(19):1854–63. 23062541 10.1016/j.jacc.2012.02.093

[3] Bekeredjian R, Grayburn PA. Valvular heart disease: aortic regurgitation. Circulation. 2005;112(1):125–34. 15998697 10.1161/CIRCULATIONAHA.104.488825

[4] Katz AM, Rolett EL. Heart failure: when form fails to follow function. Eur Heart J. 2016;37(5):449–54. 26497163 10.1093/eurheartj/ehv548

[5] Vahanian A, Beyersdorf F, Praz F, Milojevic M, Baldus S, Bauersachs J, et al. 2021 ESC/EACTS guidelines for the management of valvular heart disease. Eur Heart J. 2022;43(7):561–632. 34453165 10.1093/eurheartj/ehab395

[6] Seldrum S, de Meester C, Pierard S, Pasquet A, Lazam S, Boulif J, et al. Assessment of left ventricular reverse remodeling by cardiac MRI in patients undergoing repair surgery for severe aortic or mitral regurgitation. J Cardiothorac Vasc Anesth. 2019;33(7):1901–11. 30583928 10.1053/j.jvca.2018.11.013

[7] Villari B, Sossalla S, Ciampi Q, Petruzziello B, Turina J, Schneider J, et al. Persistent diastolic dysfunction late after valve replacement in severe aortic regurgitation. Circulation. 2009;120(23):2386–92. 19933939 10.1161/CIRCULATIONAHA.108.812685

[8] Krayenbuehl HP, Hess OM, Monrad ES, Schneider J, Mall G, Turina M. Left ventricular myocardial structure in aortic valve disease before, intermediate, and late after aortic valve replacement. Circulation. 1989;79(4):744–55. 2522356 10.1161/01.cir.79.4.744

[9] Azevedo CF, Nigri M, Higuchi ML, Pomerantzeff PM, Spina GS, Sampaio RO, et al. Prognostic significance of myocardial fibrosis quantification by histopathology and magnetic resonance imaging in patients with severe aortic valve disease. J Am Coll Cardiol. 2010;56(4):278–87. 20633819 10.1016/j.jacc.2009.12.074

[10] Puls M, Beuthner BE, Topci R, Vogelgesang A, Bleckmann A, Sitte M, et al. Impact of myocardial fibrosis on left ventricular remodelling, recovery, and outcome after transcatheter aortic valve implantation in different haemodynamic subtypes of severe aortic stenosis. Eur Heart J. 2020;41(20):1903–14. 32049275 10.1093/eurheartj/ehaa033 PMC7242071

[11] Murashita T, Schaff HV, Suri RM, Daly RC, Li Z, Dearani JA, et al. Impact of left ventricular systolic function on outcome of correction of chronic severe aortic valve regurgitation: implications for timing of surgical intervention. Ann Thorac Surg. 2017;103(4):1222–8. 27863733 10.1016/j.athoracsur.2016.09.004

[12] Herrmann S, Fries B, Salinger T, Liu D, Hu K, Gensler D, et al. Myocardial fibrosis predicts 10-year survival in patients undergoing aortic valve replacement. Circ Cardiovasc Imaging. 2018;11(8):e007131. 30354492 10.1161/CIRCIMAGING.117.007131

[13] Maznyczka A, Prendergast B, Dweck M, Windecker S, Généreux P, Hildick-Smith D, et al. Timing of aortic valve intervention in the management of aortic stenosis. JACC: Cardiovasc Interventions. 2024;17(21):2502–14. 10.1016/j.jcin.2024.08.046 39537272

[14] Arnold JR, McCann GP. Cardiovascular magnetic resonance: applications and practical considerations for the general cardiologist. Heart. 2020;106(3):174–81. 31826937 10.1136/heartjnl-2019-314856

[15] Li L, Gao J, Liu X, Chen BX, Su P, Xie B. Tissue-level evidence of fibroblast activation protein inhibitor imaging in hypertrophic obstructive cardiomyopathy: a case series. Eur Heart J. 2024;8(5):ytae189. 10.1093/ehjcr/ytae189 PMC11071445 38711681

[16] Messroghli DR, Moon JC, Ferreira VM, Grosse-Wortmann L, He T, Kellman P, et al. Clinical recommendations for cardiovascular magnetic resonance mapping of T1, T2, T2* and extracellular volume: A consensus statement by the society for cardiovascular magnetic resonance (SCMR) endorsed by the European association for cardiovascular imaging (EACVI). J Cardiovasc Magn Reson. 2017;19(1):75. 28992817 10.1186/s12968-017-0389-8 PMC5633041

[17] von Stumm M, Petersen J, Sinn M, Holst T, Sequeira-Gross TM, Muller L, et al. Correlation of myocardial native T1 and left ventricular reverse remodeling after valvular surgery. J Clin Med. 2023. . 10.3390/jcm12072649 10.3390/jcm12072649 PMC10095140 37048732

[18] Higuchi T, Serfling SE, Leistner DM, Speer T, Werner RA. FAPI-PET in cardiovascular disease. Semin Nucl Med. 2024. 10.1053/j.semnuclmed.2024.02.006. 38519308 10.1053/j.semnuclmed.2024.02.006

[19] Song W, Zhang X, He S, Gai Y, Qin C, Hu F, et al. (68)Ga-FAPI PET visualize heart failure: from mechanism to clinic. Eur J Nucl Med Mol Imaging. 2023;50(2):475–85. 36269382 10.1007/s00259-022-05994-4

[20] Settelmeier S, Kessler L, Varasteh Z, Mahabadi AA, Michel L, Papathanasiou M, et al. FAPI PET imaging supports clinical decision making in academic cardiology practice: A pictorial imaging vignette. JACC Cardiovasc Imaging. 2024;17(7):811–23. 38819338 10.1016/j.jcmg.2024.04.003

[21] Wang L, Wang Y, Wang J, Xiao M, Xi XY, Chen BX, et al. Myocardial activity at (18)F-FAPI PET/CT and risk for sudden cardiac death in hypertrophic cardiomyopathy. Radiology. 2023;306(2):e221052. 36219116 10.1148/radiol.221052

[22] Xie B, Wang J, Xi XY, Guo X, Chen BX, Li L, et al. Fibroblast activation protein imaging in reperfused ST-elevation myocardial infarction: comparison with cardiac magnetic resonance imaging. Eur J Nucl Med Mol Imaging. 2022;49(8):2786–97. 34984503 10.1007/s00259-021-05674-9

[23] Shi X, Lin X, Huo L, Li X. Cardiac fibroblast activation in dilated cardiomyopathy detected by positron emission tomography. J Nucl Cardiol. 2022;29(2):881–4. 32803676 10.1007/s12350-020-02315-w

[24] Diekmann J, Neuser J, Rohrich M, Derlin T, Zwadlo C, Koenig T, et al. Molecular imaging of myocardial fibroblast activation in patients with advanced aortic stenosis before transcatheter aortic valve replacement: a pilot study. J Nucl Med. 2023;64(8):1279–86. 37290793 10.2967/jnumed.122.265147

[25] Glantschnig L, Reitsam NG, Kircher Md M, Segmiller D, Lapa C, Dierks A. Intense FAP expression of ovarian metastatic breast cancer detected by [ 68 Ga]RTX-1363 PET/CT. Clin Nucl Med. 2025;50(5):444–5. 39847861 10.1097/RLU.0000000000005689

[26] Pan T, Einstein SA, Kappadath SC, Grogg KS, Lois Gomez C, Alessio AM, et al. Performance evaluation of the 5-Ring GE discovery MI PET/CT system using the National electrical manufacturers association NU 2-2012 standard. Med Phys. 2019;46(7):3025–33. 31069816 10.1002/mp.13576 PMC7251507

[27] El-Andari R, Watkins AR, Fialka NM, Kang JJH, Bozso SJ, Hassanabad AF, et al. Minimally invasive approaches to mitral valve surgery: where are we now? A narrative review. Can J Cardiol. 2024;40(9):1679–89. 38552791 10.1016/j.cjca.2024.03.017

[28] Zito F, Veen KM, Melina G, Lansac E, Schafers HJ, de Kerchove L, et al. Aortic valve repair in adults: long-term clinical outcomes and echocardiographic evolution in different valve repair techniques. Eur J Cardiothorac Surg. 2025. 10.1093/ejcts/ezaf020. 39871613 10.1093/ejcts/ezaf020 PMC11879640

[29] Unger P, Galloo X, Pibarot P. Mixed valvular heart disease: diagnosis and management. Eur Heart J. 2025;46(24):2261–74. 40036874 10.1093/eurheartj/ehaf116

[30] Quintana E, Suri RM, Thalji NM, Daly RC, Dearani JA, Burkhart HM, et al. Left ventricular dysfunction after mitral valve repair–the fallacy of normal preoperative myocardial function. J Thorac Cardiovasc Surg. 2014;148(6):2752–60. 25173130 10.1016/j.jtcvs.2014.07.029

[31] Alashi A, Khullar T, Mentias A, Gillinov AM, Roselli EE, Svensson LG et al. Long-Term Outcomes After Aortic Valve Surgery in Patients With Asymptomatic Chronic Aortic Regurgitation and Preserved LVEF: Impact of Baseline and Follow-Up Global Longitudinal Strain. JACC: Cardiovascular Imaging. 2020;13(1, Part 1):12–21. 10.1016/j.jcmg.2018.12.021 30772216

[32] Nestle U, Kremp S, Schaefer-Schuler A, Sebastian-Welsch C, Hellwig D, Rübe C, et al. Comparison of different methods for delineation of ^18^F-FDG PET-Positive tissue for target volume definition in radiotherapy of patients with Non-Small cell lung cancer. J Nucl Med. 2005;46(8):1342–8. 16085592

