# Supplementary material for: FAPi-PET/CT as surrogate marker for subclinical myocardial fibrosis is a prognostic tool for patients undergoing left-sided heart valve surgery
Source: EJNMMI Res. 2026 Apr 3;16:63. doi: 10.1186/s13550-025-01324-5 (PMC13076814; doi:10.1186/s13550-025-01324-5)
Supplement: Supplementary file 1 — Supplementary Material 1 [file 13550_2025_1324_MOESM1_ESM.pdf]

Cover Letter:

Prof. Dr. Dr. Ralph A. Bundschuh

Department of Nuclear Medicine
University Hospital Carl Gustav Carus Dresden, Germany

Dresden, August 11th 2025

Dear Irene,

please find attached our revised manuscript "**FAPi-PET/CT as surrogate marker for subclinical myocardial fibrosis is a prognostic tool for patients undergoing left-sided heart valve surgery**", in which we included our changes based on the reports of the two reviewers. As discussed in the point-to-point reply, which you find attached, we did not find the opportunity to do an analysis using the 17-segment AHA model. In addition, we cannot included comparison with MRI data as suggested by reviewer 1, as in the clinical workflow in Augsburg, no MRI examination was performed in these patients before surgery.
All other questions could be answered and we did an additional analysis of the right ventricle which we included in the revised manuscript.

Therefore, we hope you will find it now suitable for publication. However, if there are further questions, we are happy to answer them.

Best regards,

Ralph
