# Supplementary material for: FAPi-PET/CT as surrogate marker for subclinical myocardial fibrosis is a prognostic tool for patients undergoing left-sided heart valve surgery
Source: EJNMMI Res. 2026 Apr 3;16:63. doi: 10.1186/s13550-025-01324-5 (PMC13076814; doi:10.1186/s13550-025-01324-5)
Supplement: Supplementary file 3 — Supplementary Material 3 [file 13550_2025_1324_MOESM3_ESM.docx]

**SUPPLEMENTARY TABLES**

| **Patient** | **preoperative NT-proBNP (ng/ml)** | **preoperative LVEF (%)** | **postoperative LVEF (%)** | **SUVmax** | **SUVmean (Isocontour-55%)** | **FAP-positve volume (Isocontour-55%) (ccm)** |
| --- | --- | --- | --- | --- | --- | --- |
| 1 | 11,400 | 55 | n.a. | 3.96 | 2.51 | 130.0 |
| 2 | 2,740 | 50 | 65 | 5.91 | 3.96 | 140.5 |
| 3 | 749 | 70 | 50 | 11.25 | 7.83 | 22.9 |
| 4 | 275 | 60 | 55 | 3.94 | 2.48 | 37.2 |
| 5 | 146 | 60 | 60 | 5.21 | 3.39 | 83.6 |
| 6 | 1,680 | 68 | 60 | 6.20 | 4.08 | 95.6 |
| 7 | 131 | 60 | 55 | 6.84 | 4.44 | 102.7 |
| 8 | 697 | 50 | 44 | 4.83 | 3.05 | 106.9 |
| 9 | 1,480 | 35 | n.a. | 2.97 | 1.86 | 100.0 |
| 10 | 276 | 48 | 55 | 7.97 | 5.17 | 83.0 |
| 11 | 111 | 64 | 52 | 5.11 | 3.29 | 58.0 |
| 12 | 8,960 | 40 | 40 | 9.56 | 6.08 | 158.7 |
| 13 | 3,690 | 40 | 48 | 10.88 | 6.99 | 86.8 |

**SUPPLEMENTARY TABLE 1**: Individual patient characteristics and results.
