# Supplementary material for: FAPi-PET/CT as surrogate marker for subclinical myocardial fibrosis is a prognostic tool for patients undergoing left-sided heart valve surgery
Source: EJNMMI Res. 2026 Apr 3;16:63. doi: 10.1186/s13550-025-01324-5 (PMC13076814; doi:10.1186/s13550-025-01324-5)
Supplement: Supplementary file 2 — Supplementary Material 2 [file 13550_2025_1324_MOESM2_ESM.docx]

**Point-by-point reply**

Reviewer reports:

**Reviewer #1**: The authors investigated patients scheduled for aortic and/or mitral valve surgery which received preoperative FAPI-targeted PET. They report on associations between preoperative LVEF, preoperative NT-proBNP and LVEF change during follow-up.

Some comments:
-Introduction should be more straightforward. This reads like an interesting review article in its current form. For instance, the part on MRI could be removed or shortened, as this manuscript is dealing with PET.

Thank you very much for this input, we adapted the Introduction as suggested and shortened especially the part on MRI.

-For the section on radiopharmaceutical production, image analysis and operation, respective references are needed.

We included references about another application of the tracer, the PET/CT scanner used as well as operation techniques.

-What was the level of experience of all readers?

Both readers were board certified Nuclear Medicine specialist, one with more than five years and the other with more than three years of experience in analysis of cardiovascular PET studies. We included this information in the revised manuscript.

-Can the authors provide respective 17-AHA Segment Models? It would be interesting to see the pattern in different segments and if there are segments which show higher uptake relative to others.

This is very interesting and important questions. Unfortunately, we do not have a software package available, that was capable of matching the FAP emission data into a 17-segment model. Therefore, unfortunately, we are not capable of providing these data.

-Are there also cardiac MRI data available? A head-to-head comparison with FAPI-PET would be of interest. TTE was the method of choice to determine LVEF, but there may be respective MRIs available.

We completely agree with the reviewer, that this would be a very good comparison, and if MRI data would have been available, we would have included it in the analysis for sure. Unfortunately, for our institution, the MRI capacity is very limited, so that only one patient of the cohort does have an external MRI. For the remaining patients, unfortunately no MRI was performed. We included this point into the limitation section.

-p. 11, Discussion "Such patients would also show low FAP-expression and simultaneously lack the potential for LV functional recovery postoperatively. This statement is further supported by the previously published evidence." This should be further explained.

Thank you very much for this remark, we added two more sentences in this paragraph explain the process.

-The authors may also discuss the FAPI uptake in those two deceased patients.

In the Results section we already report, that the uptake was just moderately increased (SUVmax 4 and 3) but with a high FAP-positive volume. One may speculate, that large parts of the left ventricular myocardium is remodeled and the low uptake suggest, that the remodeling is not active any more but already far advanced. Corresponding to this in one of these patients the preoperative LVEF was already reduced with 35 %. However, in the other patient the preoperative LVEF was normal (55 %). We included a discussion of this topic in the revised manuscript.

-Figs.: Can the authors provide respective transaxial PET/CTs of the myocardium, e.g., for the three cases with heterogeneous uptake findings,

Thank you very much for this remark. We added transaxial fused PET/CT images for the three cases in Figure 1.

-Can the authors also evaluate the right ventricle?

This is a very interesting question. As you can see in our three example patients in Fig. 1, no relevant uptake can be seen in the right ventricle. This was the same for the remaining 10 patients as well. Additionally, we evaluated the uptake in the right ventricle by placing a VOI around it not including the septum. The maximum SUV values were significantly lower than the values in the left ventricle (mean 3.1 compared to 6.5 in LV). We included this information in the revised manuscript.

Minor:
-p.11, l. 4: "did not show any significant correlation" should not be repeated. Pls. rephrase.

Thank you for this remark, we rephrased the sentence accordingly.

**Reviewer #2**: Thank you for providing this interesting manuscript entitled 'FAPi-PET/CT as surrogate marker for subclinical myocardial fibrosis is a prognostic tool for patients undergoing left-sided heart valve surgery'. The authors investigated the potential of preoperative FAP-based PET imaging as a prognostic tool for patients with valvular heart disease.

Thank you very much for these positive comments.

In general, the paper is well written and concise. The authors state that a 55% SUV and a 3.5 SUVmax-based threshold isocontour was used for the FAP-positive volume. However, there is currently no established total volume threshold for FAP-based imaging. For instance, Diekmann et al. (<https://doi.org/10.2967/jnumed.122.265147>) utilised a FAP-positive threshold of blood pool SUVmean + 2 SD. Please clarify why especially these two thresholds were used to define the FAP-positive total volume in ccm?

Thank you very much for this important comment. As the reviewer already states, there is currently no established total volume threshold for FAP-based imaging, especially not for cardiac applications. Indeed, we tried the method by Diekmann et al. including the blood pool to establish a threshold as well but did find, that this led to threshold which did not lead to a volume segmentation in four of our 13 patients, therefore we decided not to use this method further. Instead we used a method based on variable and a fixed threshold using empirical threshold parameters, which are typical methods for segmenting PET data. We included this information in the PET image analysis part as well as in the discussion.
